# Virus Subverts Cellular Defense for Reproduction and Escape

**DOI:** 10.1371/journal.pbio.0030195

**Published:** 2005-04-26

Against the constant threat of infection by bacteria or viruses, one line of defense for the eukaryotic cell is the autophagosome. This double-membrane structure, which buds off from the endoplasmic reticulum, traps cytoplasmic intruders and, upon maturation, merges with a lysosome to destroy them. In this issue, however, Karla Kirkegaard and colleagues show that for one class of viruses, the autophagosome is not a holding cell but a breeding ground, and may even provide a novel escape route.

The viruses in question are picornaviruses, which include polioviruses and rhinoviruses. Infection of human cells with poliovirus is known to induce proliferation of double-membrane cytoplasmic vesicles that are morphologically similar to autophagosomes, but the origin and ultimate identity of these vesicles has not been resolved. To test whether these viral-laden vesicles are truly autophagosomes, the authors visualized two proteins: LC3, a specific marker for autophagosomes, and 3A, a part of the poliovirus RNA replication complex. After infection, these proteins colocalized, indicating the poliovirus was indeed within the autophagosome-like vesicles. LC3 also colocalized with LAMP1, a marker for lysosomes, indicating these vesicles mature in a manner similar to that of autophagosomes. This same effect could be induced simply by expressing two viral proteins.

All these results indicate that the virus stimulates production of vesicles that bear the traits of autophagosomes and contain the virus, but they don't indicate what the consequence is for viral replication. To determine that, the authors increased autophagosome production with two known stimulators of autophagy, tamoxifen and rapamycin. But rather than protecting the cell, this stimulation increased viral yield either 4-fold, in the case of tamoxifen, or 3-fold, for rapamycin. Conversely, inhibiting autophagosome production reduced viral yield. From these results, it seems the virus has subverted the components of the autophagy pathway for its own uses.

Inhibiting autophagosome production reduced viral yield inside the cell, but even more so outside. While they were not able to exclude other mechanisms, the authors argue that one possible explanation is that these vesicles are used by the virus to exit from the cell. Supporting this view, they produced electron micrographic images consistent with the fusion of the autophagosome with the plasma membrane and the extracellular release of its contents. This suggests that the virus, which is known to lyse cells to release new viral particles, has another, less lethal means of escape. This may increase the virus's chance of avoiding immune system detection as it infects new cells.

